# Difficulty in diagnosing the pathological nature of an acute fracture of the clavicle: a case report

**DOI:** 10.1186/1749-799X-4-21

**Published:** 2009-06-25

**Authors:** Sheraz S Malik, Saiqah Azad, Shahbaz Malik, Caroline B Hing

**Affiliations:** 1 Department of Trauma & Orthopaedics, Watford General Hospital, West Hertfordshire Hospitals NHS Trust, Watford, UK; 2 Department of Medicine, Watford General Hospital, West Hertfordshire Hospitals NHS Trust, Watford, UK; 3 Department of Trauma & Orthopaedics, Barnet General Hospital, Barnet & Chase Farm Hospitals NHS Trust, Barnet, UK

## Abstract

Fractures of the clavicle comprise between 5% to10% of all fractures. Medial clavicular fractures are uncommon and are normally caused by high-energy trauma. A low impact mechanism of injury should raise suspicion of a pathological fracture, but this case report highlights the difficulty in diagnosing the pathological nature of an acute fracture of the clavicle. We describe a patient who presented with a medial clavicular fracture after a simple fall but the fracture was diagnosed as pathological in retrospect four months after the initial presentation. We would also like to emphasise that the medial clavicle is the most frequent site of pathological fractures of the clavicle, and the possibility of an underlying pathological condition should be considered whenever a patient with a medial clavicular fracture is encountered.

## Background

The incidence of clavicular fractures in adults is 30 per 100, 000 population per year [1] and these are one of the most commonly encountered fractures in the accident & emergency (A&E) department and orthopaedic practice [2]. Most clavicular fractures are caused by a fall or direct trauma to the shoulder. The clavicle is vulnerable to pathological fractures from several causes such as neoplasm, infection and metabolic bone disease [3]. We describe a patient who presented with a medial clavicular fracture after a trivial activity, but the fracture was diagnosed as pathological in retrospect four months after the initial presentation. To the best of our knowledge, the delay that can occur between the first presentation of an acute clavicular fracture and recognition that it is in fact pathological has not been specifically highlighted previously in the literature.

## Case presentation

### Case report

**Figure 1:**
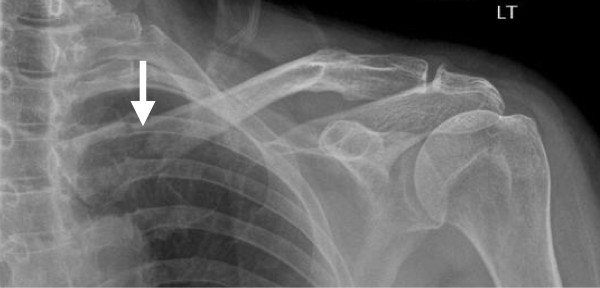
**Plain radiograph of the left shoulder at the first presentation**. The radiograph demonstrates a medial clavicular fracture (arrow) that was later diagnosed as pathological.

A 67-year old woman presented to the A&E department complaining of pain in her left shoulder and clavicle that started whilst lifting a flowerpot in the garden. She also recalled having fallen from a stepladder a few days before but denied any apparent injury resulting from this. On examination, there was swelling and tenderness over the medial aspect of the left clavicle, and no associated neurovascular deficit. The rest of the shoulder examination was normal. A plain radiograph of her left shoulder revealed an undisplaced fracture of the medial clavicle (figure 1). She was placed in a broad arm sling and discharged from the A&E department with a follow-up appointment in the fracture clinic.

One week later, the patient was reviewed in the fracture clinic by an orthopaedic registrar who attributed the pain, swelling and the fracture of the clavicle to the mechanism of injury and advised follow-up in one month's time.

**Figure 2:**
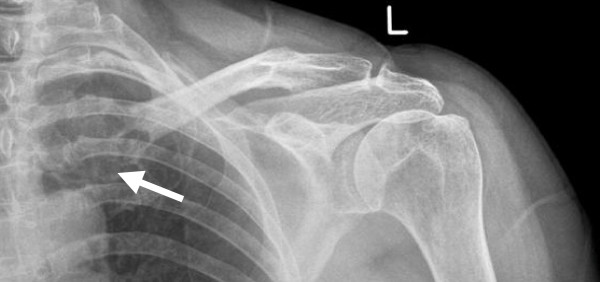
**Plain radiograph of the left shoulder taken 4 months later**. The radiograph demonstrates a large lytic lesion (arrow) over the medial aspect of the clavicle.

**Figure 3:**
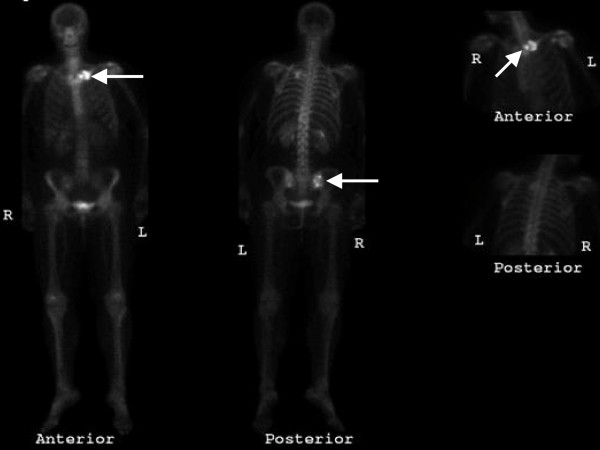
**A Tc99M MDP Bone Scan**. The bone scan demonstrates bone metastases to the left medial clavicle and the right ilium (arrows).

The patient failed to attend the follow-up appointment and was discharged from the clinic because of non-attendance. Four months later the patient was referred by the GP to the orthopaedic clinic with an enlarging lump over the fracture site. In the clinic she was systemically well with no concerning symptoms other than an enlarging swelling at the fracture site. She gave a past medical history of hypertension, a left mastectomy for breast cancer eight years ago, and admitted to smoking for many years. On examination there was a large, mildly tender bony lump at the fracture site, and a repeat radiograph of the left shoulder revealed a large lytic lesion over the medial aspect of the clavicle (figure 2). This was considered malignant and urgently investigated further. A computed tomography (CT) scan of the thorax, abdomen and pelvis revealed a right renal tumour with metastases to the lungs, liver and bone. The bone metastases were to the left clavicle and the right ilium. A further two-phase technetium-99m-methylene diphosphonate (Tc99M MDP) bone scan confirmed only two bone metastases (figure 3), and an open biopsy of the clavicle revealed a metastatic renal cell carcinoma.

The patient was referred to a medical oncologist for further staging and treatment.

### Discussion

Medial clavicular fractures are the least common of clavicular fractures, comprising between 2% to 10% of all clavicular fractures [1,4,5]. Postacchini et al found that the incidence of medial clavicular fractures increases in the elderly, comprising 2% of clavicular fractures in 18–30 years age group and 10% of clavicular fractures in 61–80 years age groups [4]. All clavicular fractures are more common in men, and in Robin's case series of 1000 clavicular fractures the male to female ratio for medial clavicular fracture was 3.7:1 [1]. Acute medial clavicular fractures are commonly caused by high-energy trauma and are associated with other multisystem injuries [5].

Renal cell carcinoma accounts for 2% of all malignancies. Up to a third of patients with renal cell carcinoma develop bone metastases [6], most of which are lytic and predominantly affect the axial skeleton [7]. Clavicular metastases comprise 6–18% of all bone metastases from renal cell carcinoma [6-8]. Swanson et al found that the symptoms secondary to bone metastases were the presenting complaint that subsequently led to a diagnosis of renal cell carcinoma in 121 of 252 (48%) patients [8]. In their study, 37 patients presented with a pathological fracture and an additional 34 patients experienced a pathological fracture in the course of the disease.

The medial clavicle is the most frequent site of pathological fractures in the clavicle [3]. A pathological fracture occurs in a bone that is not normal [9]. Failure to recognise and appropriately treat a pathological fracture and the associated underlying condition can be detrimental to the patient's life or the affected limb [9]. This case report demonstrates the difficulty in diagnosing a pathological fracture of the clavicle. The patient in this case was treated for a primary non-pathological fracture of the clavicle and the proper diagnosis was made four months after the patient's initial presentation. Clinicians are alert to suspecting a pathological fracture in unusual circumstances of injury in bones such as vertebrae and long bones of the limbs. However, clavicular fractures are common in all age groups and occur due to various types of injury and variable-energy trauma. These fractures are not routinely suspected to be pathological, unless associated with obvious clinical or radiological features of an underlying disease. Therefore clavicular fractures are not routinely investigated for an underlying pathological condition. This can result in a considerable time lag between the first presentation of the clavicular fracture and recognition that it is in fact pathological. Furthermore, an unclear history or a history of multiple accidents e.g. falls, can confound the actual cause of the fracture. In the patient that we described the fracture was attributed to the old fall but she in fact had an atraumatic pathological fracture of the clavicle.

Adeyemo et al described a similar case where a 73 year old man was seen in A&E and then followed up in a fracture clinic for a left medial clavicular fracture after a fall on to the left shoulder [10]. Six weeks after the first presentation he was found to have clinical and radiological signs of "huge callus formation" at the fracture site, and was given a 3-month follow up appointment. He was admitted to hospital with obstructive jaundice before this, and at his follow up appointment he was found to have a clavicle swelling the size of an orange and complete radiological destruction of the medial clavicle. A diagnosis of underlying metastatic bronchogenic carcinoma was later established. It was after over 4 months since first presentation that the pathological nature of the clavicular fracture was appreciated in retrospect.

To the best of our knowledge, this is the first time that the delay that can be associated with diagnosing the pathological nature of an acute clavicular fracture has been specifically brought to light. Adeyemo et al put this delay down to the "compartmentalised" treatment that their patient received from multiple health care professionals [10], but it is now emerging that this could be a feature common to acute pathological clavicular fractures as a group. Of course, many such fractures are diagnosed promptly, and may not necessarily be reported in the literature. However the delay that can occur is a significant one, four months or more in the two cases discussed above, and this has been highlighted with the aim of raising awareness in all cases.

A high index of suspicion is required to consider a clavicular fracture as pathological. For this reason, a full medical history should always be taken at the time of assessing a patient with a fracture. Information such as past medical history of carcinoma can raise a high index of suspicion of a pathological fracture. Other features in the history that could suggest a pathological fracture include a patient above the age of 45 years, multiple recent fractures or pain at the site before the fracture [9]. Such patients should also have a thorough physical examination of the upper limbs, the rest of the skeletal system to check for other affected sites, and a general examination of possible primary sites such as breast, prostate, thyroid and lymph nodes for lymphoma [9].

The clinical finding would direct further urgent investigations, and may include further imaging of the clavicle with cone views and upper rib radiographs or a CT scan to adequately delineate the fracture and the quality of the surrounding bone [11]. Adjunct investigations include laboratory studies such as full blood count, erythrocyte sedimentation rate, bone profile, prostate-specific antigen, immunoelectrophoresis and alkaline phosphatase, urine analysis for Bence-Jones proteins, CT scan of the thorax, abdomen and pelvis, total body bone/positron emission tomography (PET) scan and biopsy of the fracture site and/or the primary site if appropriate [9].

It is important that patients with a suspected pathological clavicular fracture are discussed in a multi-disciplinary setting and reviewed for features of radiographic union, unlike the patient that we described, who was discharged after failing to attend an appointment.

## Conclusion

We would like to emphasize that all patients should be carefully assessed on an individual basis, including those who present with apparently common simple injuries. We would also like to highlight that medial clavicular fractures are separate from other clavicular fractures because these are uncommon, normally associated with high-energy trauma and occur where pathological fractures in the clavicle are most common. Therefore, the possibility of an underlying pathological condition should be considered whenever a patient with a medial clavicular fracture is encountered.

## Consent

Written informed consent was obtained from the patient for publication of this case report and any accompanying images. A copy of the written consent is available for review by the Editor-in-Chief of this journal.

## Competing interests

The authors declare that they have no competing interests.

## Authors' contributions

SSM conceived the idea and wrote the paper.

SA and SM analysed the notes and contributed to the discussion.

CBH was responsible for editing and approving the final manuscript.

All authors read and approved the final manuscript.
